# Astaxanthin Prevents Alcoholic Fatty Liver Disease by Modulating Mouse Gut Microbiota

**DOI:** 10.3390/nu10091298

**Published:** 2018-09-13

**Authors:** Huilin Liu, Meihong Liu, Xueqi Fu, Ziqi Zhang, Lingyu Zhu, Xin Zheng, Jingsheng Liu

**Affiliations:** 1 School of Life Sciences, Jilin University, Changchun 130012, China; lhl14@mails.jlu.edu.cn (H.L.); fxq@jlu.edu.cn (X.F.); 2 College of Food Science and Engineering, Jilin Agricultural University, Changchun 130118, China; liumh@jlau.edu.cn; 3 National Engineering Laboratory for Wheat and Corn Deep Processing, Changchun 130118, China; 4 College of Animal Science and Technology, Jilin Agricultural University, Changchun 130118, China; zhangziqi@jlau.edu.cn (Z.Z.); zhulingyu@jlau.edu.cn (L.Z.); zhengxin@jlau.edu.cn (X.Z.)

**Keywords:** astaxanthin, *Akkermansia*, alcoholic fatty liver disease, inflammation, gut microbiota

## Abstract

The development and progression of alcoholic fatty liver disease (AFLD) is influenced by the intestinal microbiota. Astaxanthin, a type of oxygenated carotenoid with strong antioxidant and anti-inflammatory properties, has been proven to relieve liver injury. However, the relationship between the gut microbiota regulation effect of astaxanthin and AFLD improvement remains unclear. The effects of astaxanthin on the AFLD phenotype, overall structure, and composition of gut microbiota were assessed in ethanol-fed C57BL/6J mice. The results showed that astaxanthin treatment significantly relieves inflammation and decreases excessive lipid accumulation and serum markers of liver injury. Furthermore, astaxanthin was shown to significantly decrease species from the phyla Bacteroidetes and Proteobacteria and the genera *Butyricimonas*, *Bilophila*, and *Parabacteroides*, as well as increase species from Verrucomicrobia and *Akkermansia* compared with the Et (ethanol)group. Thirteen phylotypes related to inflammation as well as correlated with metabolic parameters were significantly altered by ethanol, and then notably reversed by astaxanthin. Additionally, astaxanthin altered 18 and 128 KEGG (Kyoto Encyclopedia of Genes and Genomes) pathways involved in lipid metabolism and xenobiotic biodegradation and metabolism at levels 2 and 3, respectively. These findings suggest that *Aakkermansia* may be a potential target for the astaxanthin-induced alleviation of AFLD and may be a potential treatment for bacterial disorders induced by AFLD.

## 1. Introduction

According to the “Global Status Report on Alcohol and Health 2014” released by the World Health Organization (WHO), the use of alcohol has reached a detrimental level, resulting in more than 3 million deaths every year and accounting for 5.9% of all deaths worldwide. As the metabolism of alcohol in the organism mainly depends on the liver, the long-term consumption and over-consumption of alcohol leads to liver damage and triggers alcoholic fatty liver disease (AFLD) [1], thereafter causing health issues ranging from fatty liver to alcoholic hepatitis and even cirrhosis [2,3]. The first two phases can be reversible through alcohol abstinence and lifestyle intervention. Thus, the detection of potential functional ingredients for AFLD prevention is significant.

Astaxanthin is one of the major xanthophyll carotenoids in marine organisms, and is found in shrimp, crabs, fish, algae, yeast, and feathers of birds [4,5]. As astaxanthin cannot be synthesized in humans, its uptake fully depends on dietary sources. Astaxanthin-associated protection against aging as well as cardiovascular and cancerous diseases is attributed to its great antioxidative and anti-inflammatory activity [4]. In particular, it has been suggested that astaxanthin is protective against various types of liver damage [6,7], such as non-alcoholic fatty liver disease (NAFLD) [8,9,10] and liver fibrosis [11]; however, further investigation is required to determine the effect of astaxanthin on AFLD protection.

Previous studies have confirmed that gut microbiota imbalance is related to a variety of chronic diseases, such as obesity, cardiovascular disease, and cancer [12,13,14]. The relationship between gut microbiota and AFLD has become a topic of increasing concern to researchers. In healthy humans, the intestinal microbiota remains in symbiotic balance, but alcohol intake induces the modification of its composition, thereby inhibiting dominant intestinal bacteria and promoting a small amount of pathogenic bacteria overgrowth [15], resulting in the impairment of intestinal physiological function. The long-term intake of alcohol breaks the barrier function of the intestine and promotes the growth of intestinal pathogenic bacteria and harmful metabolites (such as lipopolysaccharides). These health-threatening components lead to the development of AFLD by invading into other tissues and organs through blood circulation [16,17]. *Akkermansia muciniphila* (*A. muciniphila*), which can degrade mucin and maintain intestinal barrier integrity, colonizes in the human gut mucus layer and is negatively associated with certain diseases like obesity, diabetes, inflammation, and metabolic disorders [18]. Long-term dietary intervention can restore the gut microbiota and improve intestinal physiological function [19]. The concentration of *A. muciniphila* can be increased through the ingestion of *Bifidobacteria*, fructo-oligosaccharides, short-chain carbohydrates, metformin, rhubarb extract, or specific antibiotics [18]. Bacterial imbalance is an important factor in inducing disease in AFLD patients. An increase in probiotics species and the inhibition of pathogenic bacteria may improve AFLD status.

Astaxanthin has been reported to prevent liver damage [6,20], but there has been little research on the effect of astaxanthin on AFLD. Studying intestinal microbiota regulation in AFLD may provide new insight into the pathogenesis and therapeutic target of AFLD. In the present study, we investigated whether astaxanthin can protect against alcohol-induced liver injury and its association with the intestinal microecology. We also determined the main influence of bacteria during the astaxanthin intervention.

## 2. Materials and Methods

### 2.1. Animal Experimentation

Male C57BL/6J mice (22 ± 2 g, six weeks old) were purchased from the Beijing Vital River Laboratory Animal Technology Co., Ltd. (Beijing, China) and housed individually in cages with a 12-h light/dark cycle at 23 ± 2 °C with full access to chow diet and water. All experiment protocols were approved by the Institutional Animal Care and Use Committee at the Jilin Institute of Traditional Chinese Medicine (approval number: SYXK (JI) 2015-0009).

The alcoholic liver disease (AFLD) mouse model was established using the modified Lieber–DeCarli liquid diet [21]. The 60 mice were randomly separated into five groups, each consisting of 12 mice. The first group was fed with a normal standard growth diet while the other four groups were fed high-fat liquid diets (35% fat, 18% protein, 47% carbohydrates, provided by TROPHIC Animal Feed High-Tech Co., Ltd., Nantong, China) for an acclimation period of two weeks acclimation. Then the four high-fat diet groups were fed with either only the high-fat liquid diet (Con), or combined with the astaxanthin (AST group, 50 mg/kg bw), ethanol-containing (Et group, 5% ethanol *v*/*v*, accounted for 36% of the total caloric intake), or ethanol plus astaxanthin (EtAST group) treatments for 12 weeks. Compositions of the diets are shown in Supplementary Table S1. The astaxanthin was purchased from Sigma-Aldrich (St. Louis, MO, USA; purity ≥97%, SML0982), isolated from *Blakeslea trispora* and dissolved in corn oil for utilization in this experiment. The dose selection of astaxanthin was chosen in accordance with previous research [22], while the amount of ethanol included increased over two weeks to reach a final concentration of 5% (*v*/*v*). Body weight gain and food intake were assessed once a week. The pair-fed control group (Con) was included in this model.

After 12 weeks of treatment, the fasted mice were euthanized and blood samples were collected. The feces in the colon were also harvested into 2-mL sterile tubes to assess the gut microbiota. The tissues were immediately removed and weighed, and the liver coefficient (liver weight/body weight) was calculated. The left lobe of the liver was instantly fixed in 10% buffered formalin for histology analysis with the rest of the tissues frozen in liquid nitrogen and stored at −80 °C until further use.

### 2.2. Biochemical Analyses

The serum was obtained from the collected blood by centrifugation at 5000 rpm for 10 min at 4 °C and stored at −80 °C. Plasma triacylglycerol (TG), total cholesterol (TC), high-density lipoprotein (HDL), low-density lipoprotein (LDL), aspartate aminotransferase (AST), and alanine aminotransferase (ALT) were measured by enzymatic colorimetric assays using commercial detection kits (Nanjing Jiancheng of Bioengineering Institute, Nanjing, China).

### 2.3. Hepatic Triglyceride Staining

The left lobe of the liver tissue was separated and rapidly fixed in 4% neutral buffered formalin solution for 24 h and then processed for paraffin embedding. Five-micrometer-thick paraffin sections were stained with hematoxylin and eosin (H&E) and oil-red solution. The liver steatosis status was examined under a light microscope (Olympus, Tokyo, Japan), and photographed at 200× magnification.

### 2.4. Quantification of Genes Expression in Liver Tissue

Total RNA was extracted and purified in the liver tissue using TRIzol reagent (TAKARA, Beijing, China. RNAiso Plus, Code No. 9108). The purity, concentration, and quality of RNA were measured. High-quality RNA was converted to cDNA using PrimeScript™ RT reagent Kit with gDNA Eraser (TAKARA, Beijing China. Code No. RR047A). The SYBR fluorescent dye method (TAKARA, Beijing China. Code No. RR420A) and Agilent Stratagene Mx3000P Real-Time PCR System (Santa Clara, CA, USA) were used to detect the gene expression. The primers used are shown in Supplementary Table S2. The data were calculated using the 2^−ΔΔ*C*t^ relative quantification method and normalized to β-actin.

### 2.5. Fecal DNA Extraction

Total bacterial genomic DNA was extracted from the fecal material in the colon using Fast DNA SPIN extraction kits (MP Biomedicals, Santa Ana, CA, USA), following the manufacturer’s instructions. The DNA yield was measured for quantity using a NanoDrop ND-1000 spectrophotometer (Thermo Fisher Scientific, Waltham, MA, USA) and the quality was analyzed by 0.8% agarose gel electrophoresis; the DNA was then stored at −20 °C until further analysis.

### 2.6. Amplification and Sequencing of the 16S rRNA Genes

The V3–V4 region of bacterial 16S rRNA genes was subjected to PCR amplification using the forward primer 5′-ACTCCTACGGGAGGCAGCA-3′ and the reverse primer 5′-GGACTACHVGGGTWTCTAAT-3′. The specific system was a 25-µL reaction including 5 µL of Q5 High-Fidelity DNA Polymerase (New England Biolabs (Beijing) Ltd., Beijing, China). PCR amplicons were purified with Agencourt AMPure Beads (Beckman Coulter, Indianapolis, IN, USA) and quantified using the PicoGreen dsDNA Assay Kit (Invitrogen, Carlsbad, CA, USA). The amplicons were pooled and normalized, and then paired-end 2 × 300 bp sequencing was performed using the Illumina MiSeq platform with the MiSeq Reagent Kit v3 at Shanghai Personal Biotechnology Co., Ltd. (Shanghai, China).

Sequencing data for the 16S rRNA sequences have been deposited in the SRA database under GenBank accession NO. SRP148082.

### 2.7. Bioinformatics and Statistical Analysis

Sequence data were processed using Quantitative Insights into Microbial Ecology (QIIME, v1.8.0), as previously described [23]. The low-quality sequences, which had lengths of <150 bp and average Phred scores of <20, and contained ambiguous bases and mononucleotide repeats of >8 bp, were filtered with the following criteria. Paired-end reads were assembled using FLASH. The remaining high-quality sequences were clustered into operational taxonomic units (OTUs) at 97% sequence identity by UCLUST (Edgar 2010) [24]. OTU taxonomic classification was conducted by BLAST and the OTUs containing more than 99.999% of total sequences across all samples were reserved.

All results are presented as means ± standard deviation. Data were analyzed with SPSS 19.0 using one-way analysis of variance (ANOVA), and, when appropriate, using a two-tailed Student’s *t*-test between different groups. Differences among groups were evaluated for significance with the comparable variances, followed by Tukey’s and least significant difference (LSD) tests. *p* < 0.05 was considered statistically significant. GraphPad Prism 7.0 software (GraphPad Software, La Jolla, CA, USA) was used for graph-making.

Sequence data analysis was mainly performed using QIIME (v1.8.0, University of Colorado, Denver, CO, USA) and R packages (v3.2.0, Bell Labs Technology Showcase, Murray Hill, NJ, USA). The alpha diversity indices, Chao1 richness estimator, and the Shannon diversity index were calculated using the OTU table in QIIME. Beta diversity analysis was performed using UniFrac distance metrics and visualized by principle coordinate analysis (PCoA), and the unweighted pair-group method with arithmetic means (UPGMA) hierarchical clustering. The significance of microbiota structure differentiation among groups was assessed by PERMANOVA (permutational multivariate analysis of variance) and ANOSIM (analysis of similarities) using the R package “vegan”. Taxa abundances at different taxonomies were statistically compared among groups by Metastats and visualized as box plots. Microbial functions were predicted by PICRUSt (phylogenetic investigation of communities by reconstruction of unobserved states), based on high-quality sequences.

## 3. Results

### 3.1. Astaxanthin Protects Mice from High-Fat Diet and Ethanol-Induced Liver Lesions

**Figure 1:**
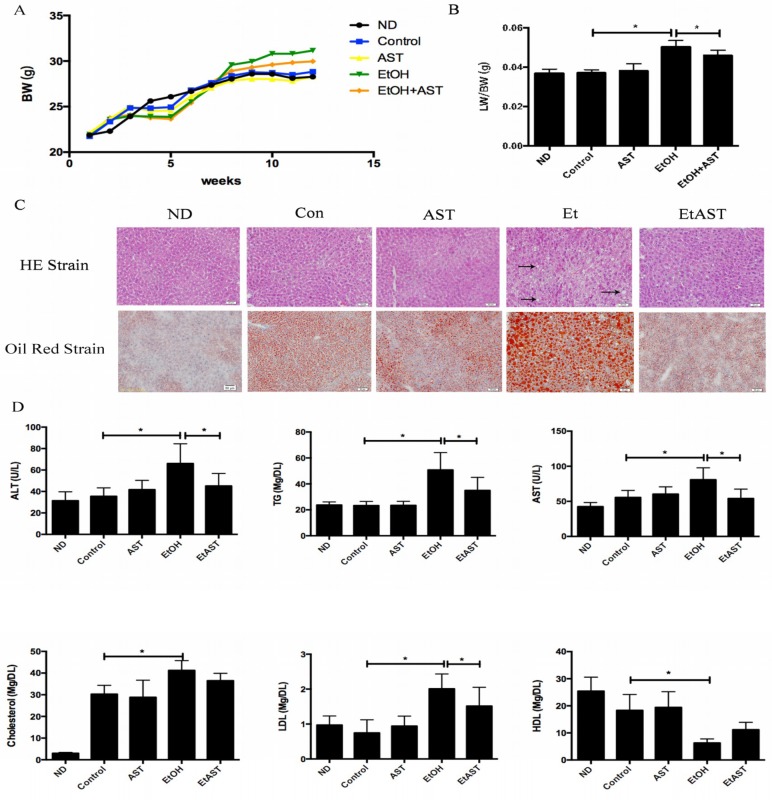
Effect of astaxanthin on body weight, pathological morphology, and serum markers involved in liver injury in ethanol diet-fed mice. C57BL/6J mice were fed the Lieber–DeCarli liquid diet containing 5% ethanol for 12 weeks ad libitum with or without 50 mg kg^−1^ of astaxanthin. Body weight was measured once a week (**A**). The liver index was represented by calculating liver weight/body weight (**B**). The statues of hepatic steatosis were checked by hematoxylin and eosin (H&E) staining and hepatic lipid accumulation was detected by Oil red O staining. The symbol → plays a indicate role to identify the fat vacuoles (**C**). The serum markers of liver injury were determined by the enzyme activities of alanine aminotransferase (ALT) and aspartate aminotransferase (AST). Lipid accumulation in the liver was reflected by the levels of hepatic markers, including plasma triacylglycerol (TG), low-density lipoprotein (LDL), high-density lipoprotein (HDL), and total cholesterol (TC) (**D**). ND: normal diet; Con: high-fat diet with 35% of total calories from fat; AST: high-fat diet + astaxanthin; Et: Lieber–DeCarli liquid ethanol diet with 35% of total calories from fat; EtAST: Lieber–DeCarli liquid ethanol diet with 35% of total calories from fat + astaxanthin. All values represent means ± SD. * p < 0.05 represents significant differences in each group compared with the ethanol (Et) group by ANOVA analysis.

We investigated whether different diets affect the growth status of mice. The five groups have similar body weights at baseline which were still not significantly difference after 12 weeks of intervention (Figure 1A). On the other hand, compared with the Control (Con) group, the liver indices of mice in the ethanol group were significantly increased, and markedly reversed in the EtAST (ethanol plus astaxanthin treatments) group, which was not different from that of the Con group (Figure 1B). The light microscopy images of the liver slices, stained by Oil Red O, showed that lipid droplets increased in size and number in the Et group compared with those in the Con group. Astaxanthin intervention significantly relieved fat accumulation in the liver induced by ethanol. H&E staining showed the structure of hepatic lobules and neatly arranged normal liver cells in the normal diet (ND) group, which showed no obvious differences among the Con and AST groups. Ethanol supplementation markedly increased the amount of hepatic steatosis and the number of necrotic cells, which appeared in a large number of fat vacuoles within liver cells and significantly enlarged them. Hepatic steatosis caused by ethanol was reversed by astaxanthin intervention (Figure 1C).

There were no significant changes in the serum markers involved in lipid dysmetabolism and liver injury in normal and astaxanthin-supplied groups compared with those in the Con group. However, the levels of ALT and AST were markedly increased in the Et group compared to the Con group, indicating the existence of liver damage, which affected cell membrane permeability and promoted ALT and AST overflow, suggesting that mice suffered significant alcoholic liver injuries. Moreover, 50 mg kg^−1^ astaxanthin treatment significantly decreased the levels of ALT, AST, TG, and LDL compared to the Et diet. However, there were no obvious differences in the levels when compared with the ND group. These results suggest that astaxanthin has the ability to alleviate lipid dysmetabolism and alcohol-induced liver injury (Figure 1D).

### 3.2. Astaxanthin Can Relieve Liver Injury Through the Regulation of Inflammatory Genes Expression in Mice

**Figure 2:**
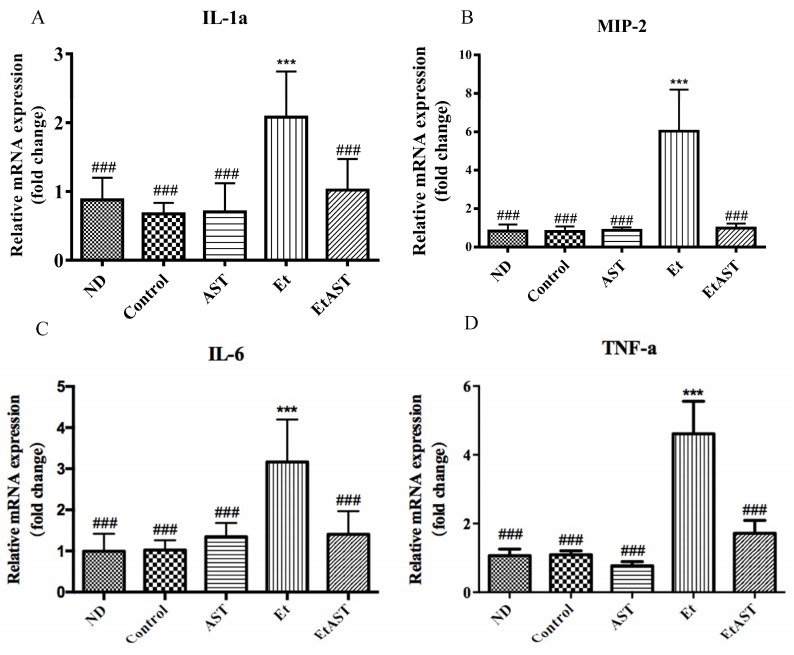
Effect of astaxanthin on inflammatory genes expression in alcoholic fatty liver disease (AFLD) mice. At the end of the experiment, total RNA was extracted from liver tissues. The mRNA expressions of interleukin-1 alpha (IL-1α) (**A**), macrophage inflammatory protein 2 (MIP-2) (**B**), interleukin-6 (IL-6) (**C**), and tumor necrosis factor-alpha (TNF-α) (**D**) were normalized to that of β-actin. Data are presented as means ± SD, *n =* 6. *** *p* < 0.001 compared with the control (Con) group, and ^###^ *p* < 0.001 compared with the Et group.

To explore whether astaxanthin can reverse the development of AFLD that is associated with inflammatory responses, liver inflammatory gene expression in liver was measured. A high-fat diet did not promote inflammatory gene expression. However, as expected, the consumption of ethanol in a high-fat diet significantly induced the mRNA expression of interleukin-1 alpha (IL-1α), macrophage inflammatory protein 2 (MIP-2), interleukin-6 (IL-6), and tumor necrosis factor-alpha (TNF-α). However, these effects were markedly reversed by astaxanthin supplementation (Figure 2).

### 3.3. Astaxanthin Alters the Profiles of Gut Microbiota in Ethanol-Fed Mice

The microbiota can influence the development and progression of AFLD. High-throughput 16S rRNA gene sequencing produced a total of 332,981 good-quality sequences from 15 samples (with 21,532 ± 231 sequences per sample) (Table S3). Rarefaction curves and rank abundance curves have shown that the most gut microbes in samples were captured based on the current sequencing depth and the data can be used for further analysis (Figure S1).

**Figure 3:**
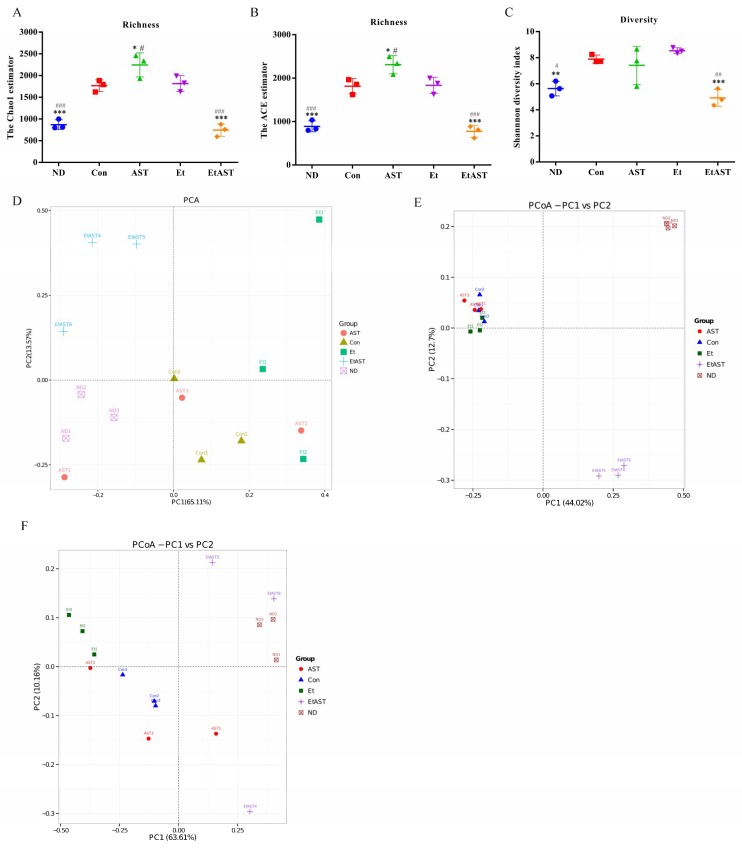
Astaxanthin treatment ameliorates the microbiota profiles affected by the ethanol diet. Bacterial genomic DNA was extracted from the feces collected at the end of week 12, and 16S rDNA sequence analysis was performed. Alpha diversity analysis, the Chao1 estimator (**A**), the ACE estimator (**B**), and the Shannon diversity index (**C**) were used for evaluation. To investigate the similarity of gut microbial community structure among different samples, we conducted beta-diversity analysis and PCA analysis (**D**), PCoA score plot based on unweighted UniFrac (**E**), and weighted UniFrac (**F**). ND: normal diet; Con: high-fat diet with 35% of total calories from fat; AST: high-fat diet + astaxanthin; Et: Lieber–DeCarli liquid ethanol diet with 35% of total calories from fat; EtAST: Lieber–DeCarli liquid ethanol diet with 35% of total calories from fat + astaxanthin. ^#^ represents *p* < 0.05, ^##^ represents *p* < 0.01, and ^###^ represents *p* < 0.001 compared with the Con group, * represents *p* < 0.05, ** represents *p* < 0.01, and *** represents *p* < 0.001 compared with the Et group.

Next, we analyzed the gut bacterial community in mice affected by high-fat and/or ethanol exposure with or without astaxanthin. Alpha-diversity analysis indicated that the values of Chao1, abundance-based coverage estimator (ACE), and Shannon were significantly increased in the Con and Et groups compared to the ND group. After treatment with 50 mg kg^–1^ astaxanthin, the values were not influenced by the high-fat diet alone but markedly decreased when ethanol was added, and even improved relative to the normal group (Figure 3A–C). Furthermore, to investigate the similarities in gut microbial community structure among different samples, certain analyses were conducted. The PCoA plot indicated that the structure of gut microbiota in the Et group was statistically different from the ND along the PC1 axis (59.16% and 42.3% of overall variation based on weighted and unweighted UniFrac, respectively), and a significant structural shift was also shown for most of the astaxanthin-supplemented mice compared with those in the Et group (Figure 3D–F). As expected, the results of the unweighted pair group method with arithmetic mean (UPGMA) based on weighted and unweighted Unifrac also showed overt changes in the composition of gut microbiota in the EtAST group compared with the Et group (Figure S2), which is in line with the PCoA results, indicating that the microbial structure was disturbed by ethanol feeding, but remedied and returned to normal status by astaxanthin administration.

### 3.4. Astaxanthin Regulates the Gut Microbiota Composition in Ethanol Feeding Mice

**Figure 4:**
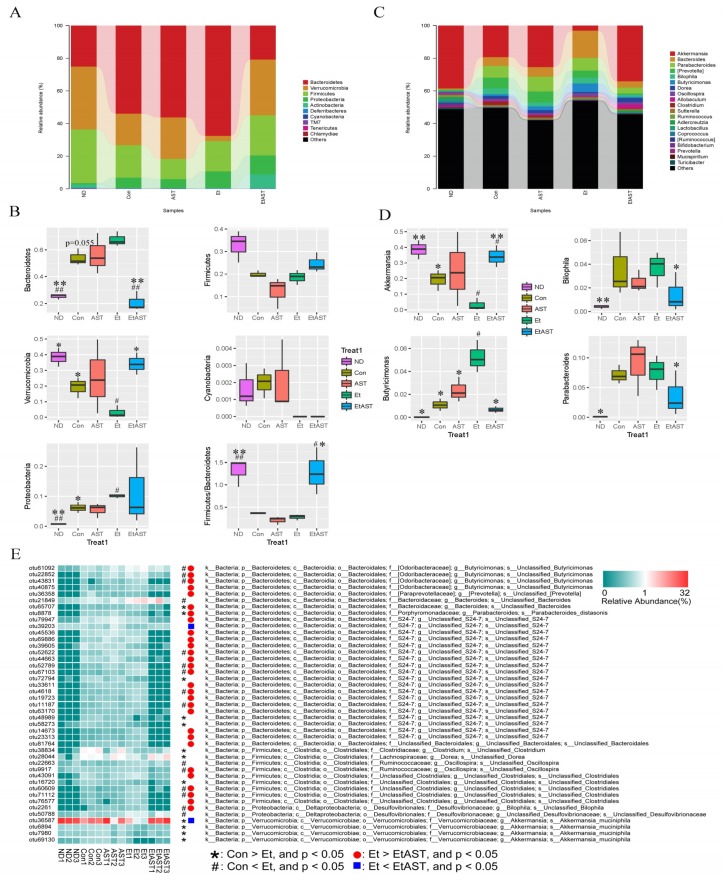
Bacterial community analysis and comparison. The composition and abundance distributions of each group at the phylum (**A**) and genus (**C**) levels were shown using QIIME software. At the phylum (**B**) and genus (**D**) levels, pairwise comparisons, conducted to determine the sequence amounts between two groups, were presented as pair-wise comparisons using Metastats analysis. ^#^ represents *p* < 0.05 and ^##^ represents *p* < 0.01 compared with the Con group; * represents *p* < 0.05 and ** represents *p* < 0.01 compared with the Et group. A heat map of 43 operational taxonomic units (OTUs) which were altered in abundance by ethanol or astaxanthin is shown, based on the redundancy analysis (RDA) model. OTUs with a relative abundance greater than 0.1% in at least in one group were selected and used to analyze these differences. The red and green colors indicate the relative abundances of OTUs that were more or less abundant. The symbols represent the OTUs whose abundance were reduced and increased in the Et group relative to the Con group, while the circles in red and rectangles in blue represent the OTUs whose abundances were reduced and increased in the EtAST group relative to the Et group (**E**).

We detected nine bacterial phyla and 60 genera among the mice. The structure and composition of gut microbiota were significantly influenced by the high-fat plus ethanol diet. The results of the top 20 most abundant OTUs at all taxonomic levels in samples, as inferred by GraPhlAn, showed that *Firmicutes*, *Bacteroidetes*, *Proteobacteria*, and *Verrucomicrobia* were the most abundant phyla, and *Akkermansia*, *Bacteroides*, *Prevotella*, and *Paraprevotella* were the most abundant genera among the OTUs (Figure S3A). Additionally, the relative bacterial abundance in groups was reflected by the cladogram and linear discriminant analysis (LDA) score (Figure S3B,C). The taxonomic profiles indicated that the proportions of *Bacteroidetes* and *Proteobacteria* increased significantly, and the abundance of *Verrucomicrobia* decreased markedly in the Con group, especially in the Et group when compared with those of the ND group, while astaxanthin was shown to significantly reverse the tendency of this bacterial abundance, similar to the ND group (Figure 4A,B). Specifically, *Cyanobacteria* was completely depleted in both the Et and EtAST groups (Figure 4B). At the genus level, bacterial taxa displayed obvious changes in the heat maps, which were affected by different diets. The genera abundance, including *Akkermansia*, *Bacillus*, *Adlercreutzia*, *Lactococcus*, *Bacteroides*, *Butyricimonas*, *Parabacteroides*, and *Bilophila*, was significantly switched by ethanol feeding compared with the normal diet and partially reversed by the addition of astaxanthin (Figure S4). Astaxanthin treatment significantly decreased the *Butyricimonas*, *Bilophila*, and *Parabacteroides* concentrations relative to the Et group, and the abundance of *Akkermansia* decreased markedly in the Et group (3%) and recovered dramatically in the EtAST group (34%), while the abundance was similar to that of the ND group (38%, Figure 4D and Figure S5)*.*

Next, we identified changes in the strain-specific key phenotypes which were affected by astaxanthin in the AFLD mice. The results showed that the abundance levels of 27 OTUs were markedly changed by ethanol supplementation (15 increased and 12 decreased OTUs) compared with those of the Con group, while astaxanthin intervention significantly altered 31 OTUs including enhanced or reduced abundance in 2 and 29 OTUs, respectively (EtAST group vs. Et group). Particularly, among the 43 OTUs, 13 were significantly increased or decreased by ethanol, and afterwards notably reversed by astaxanthin (Figure 4E), which including bacteria belonging to *Akkermansia_muciniphila*, species from *Butyricimonas*, *S24-7*, *Oscillospira*, *Clostridiales*, and *Bilophila.*

### 3.5. Associations of the Bacterial Abundance Altered by Astaxanthin with the AFLD Phenotype

**Figure 5:**
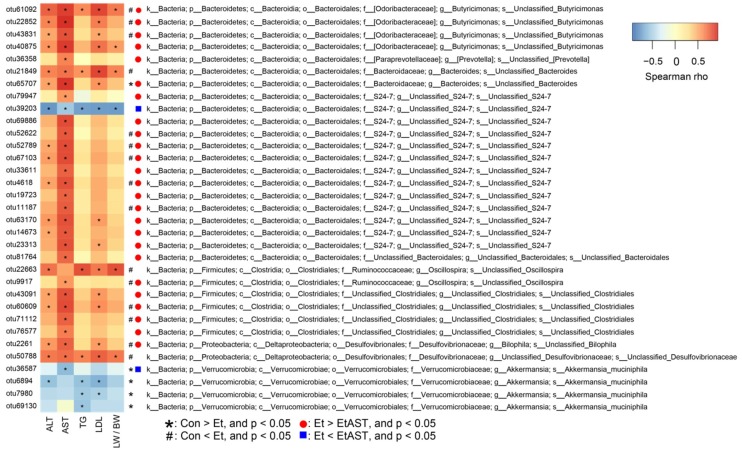
Heat map of 33 OTUs which were significantly associated with the AFLD disease phenotype altered by ethanol or astaxanthin as determined by Spearman’s correlation coefficient. These 33 OTUs were selected from the 43 OTUs which had significant changes after ethanol or astaxanthin treatment. The red and blue colors indicate the relative abundance of OTUs that were more or less abundant. The symbols * and ^#^, shown on the right-hand side of the map, represent the OTUs whose abundances were reduced and increased in the Et group relative to those of the Con group, while the red circles and blue rectangles represent the OTUs whose abundances were reduced and increased in the EtAST group relative to the Et group. The symbol * in the cells of the heat map represents the significant correlation between the corresponding metabolic parameters and OTU abundance.

To assess the relationships between OTUs and metabolic parameters altered by astaxanthin, Spearman’s correlation coefficient was employed. Among the 43 OTUs that were altered in abundance by ethanol or astaxanthin shown in Figure 5, 33 OTUs were markedly correlated with at least one of the following metabolic parameters: AST, ALT, TG, LDL, liver weight/body weight (LW/BW). Thirty-eight of these OTUs were positively correlated with the abnormal parameters, and five OTUs were negatively correlated with abnormal parameters. The abundance levels of 20 OTUs were markedly changed by ethanol supplementation (15 increased and five decreased OTUs) compared with those of the Con group, while astaxanthin intervention significantly altered 27 OTUs, including enhanced and reduced abundances in two and 25 OTUs, respectively, compared with those of the Et group. Notably, 13 OTUs were significantly increased or decreased by ethanol and then markedly reversed by astaxanthin (Figure 5).

### 3.6. Predicted Metabolic Functions of the Metagenome in Gut Microbiota

**Figure 6:**
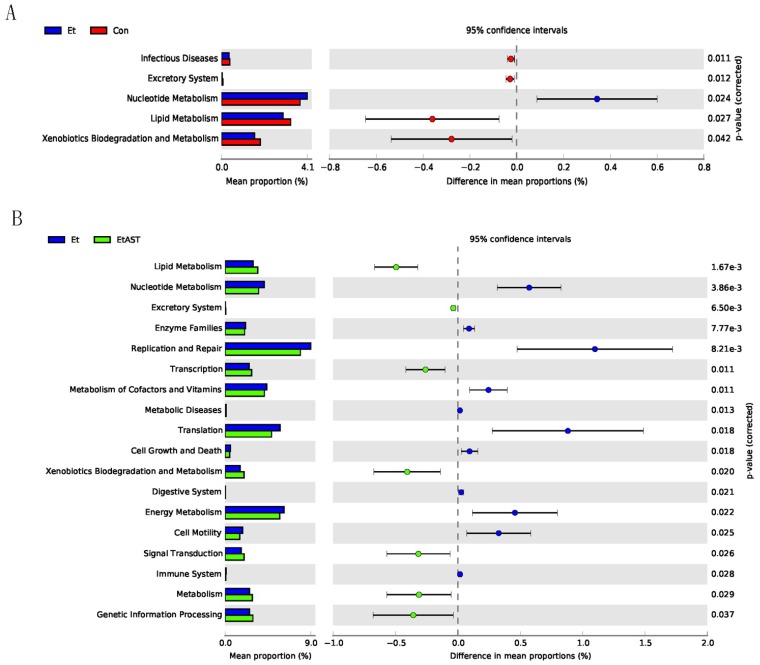
Predicted functions for the altered metagenome of gut microbiota in each group shown with KEGG (Kyoto Encyclopedia of Genes and Genomes) pathways. These data were obtained by PICRUSt. (**A**) A total of five markedly altered KEGG pathways at level 2 in the Et group compared with that in the Con group. (**B**) A total of 18 significantly changed the pathways through astaxanthin supplementation in alcoholic fatty liver disease (AFLD) mice.

The PICRUSt analysis based on metagenomes was used to predict the metabolic function of gut microbiota that were influenced by astaxanthin in AFLD mice. The results revealed that 18 and 128 KEGG (Kyoto Encyclopedia of Genes and Genomes) pathways were changed in the EtAST group at levels 2 (Figure 6B) and 3 (Figure S6), respectively, among which seven were increased and 11 were decreased compared to the Et group at level 2. In particular, we found several interesting changes among the 128 altered KEGG pathways at level 3. Firstly, the biosynthesis processes of bacteria, such as nucleotide metabolism (level 2), was increased in the Et group compared to that in the Con group, including pyrimidine metabolism, energy metabolism, DNA replication proteins, and cytoskeleton proteins (level 3), while astaxanthin intervention significantly restrained these pathways. In addition, the metagenome of the EtAST group was enriched in the pathways related to lipid metabolism, including glycerophospholipid metabolism; arachidonic acid metabolism; the biosynthesis of unsaturated fatty acids; fatty acid elongation in mitochondria; fatty acid metabolism; the synthesis and degradation of ketone bodies; xenobiotic biodegradation and metabolism, including aminobenzoate degradation, atrazine degradation, caprolactam degradation, drug metabolism, cytochrome P450 and fluorobenzoate degradation; and amino acid metabolism, including tryptophan metabolism, lysine degradation, tyrosine metabolism, and phenylalanine metabolism.

## 4. Discussion

Increasing evidence indicates that the gut microbiota could significantly affect the emergence and development of AFLD [25,26]. Astaxanthin can prevent liver injury by suppressing inflammation, fibrosis [27], and fat accumulation [10,28]. However, previously, there was little evidence suggesting an effect of astaxanthin on AFLD protection. Here, we demonstrated that astaxanthin intervention can mitigate ethanol-induced hepatic steatosis through the reconstruction of the gut microbiota structure and a subsequent increased abundance of *Akkermansia*.

Organ dysfunction is mainly associated with prolonged excessive drinking, which leads to tissue injury-related conditions, such as AFLD, with initial symptoms like fatty liver, and may be responsible for the development of alcoholic hepatitis, liver fibrosis, and cirrhosis. Proper dietary habits can reverse the pathological state of fatty liver in the early stages [29]. Astaxanthin has been proven to protect against liver injury in mice suffering from NAFLD [27] and liver fibrosis in mice [7] due to its anti-inflammation and antioxidant ability. In addition, Zheng et al. has demonstrated that astaxanthin can protect against maternal ethanol-induced embryonic developmental retardation in C57BL/6J mice [22]. In the present study, we found that astaxanthin can protect against ethanol-induced liver injury in mice by alleviating lipid accumulation, inflammatory cell infiltration, and necrosis in the liver. Furthermore, we explored whether astaxanthin could reverse the AFLD development associated with inflammatory responses, and we detected inflammatory gene expression. Various inflammatory mediators and cytokines can be generated after the activation of inflammatory signaling, particularly the major inflammatory markers TNF-α and IL-6 [30]. IL-1α and MIP-2 are important pro-inflammatory cytokines [31,32] that are significantly induced by ethanol. Astaxanthin significantly decreased these mRNA expression level and partly protected the liver from inflammation and injury.

Increasing evidence indicates that the gut microbiota is responsible for the pathogenesis and development of liver disease. A large microbial ecosystem exists in the human gastrointestinal tract, which is associated closely with health and disease control [33]. The gut–liver circulation pathway plays a critical role in alcohol metabolism and is strongly regulated by the gut microbiota. It has been proven that dietary perturbations have dominant effects on the gut microbiota [34]. We further identified bacterial groups that are significantly affected by ethanol and astaxanthin supplementation. Phyla and genera imbalance are associated with health disorders. In this model of alcohol-induced liver injury, we found that several *Bacteroidetes*, including the genera *Bacteroides*, *Butyricimonas*, and *Parabacteroides*, were significantly increased. Consistent with our results, Llopis et al. reported that after five weeks’ consumption of a diet including alcohol, *Bacteroides* was significantly more represented in severe alcoholic hepatitis mice relative to the control group [35]. Similarly, other studies have reported that the relative abundance of *Bacteroidetes* increases in alcohol-fed mice [36,37,38]. The phylum of *Bacteroidetes* is composed of three major classes of Gram-negative bacteria, normally resident in the intestines, mouth, upper respiratory tract, and genital tracts of humans and animals, which have been described as having both beneficial and detrimental features. *Bacteroidetes* can lead to endogenous infections due to a micro-ecological imbalance. In particular, *Bacteroides fragilis* can produce polysaccharide A to relieve colitis in animals [39]. However, it can also produce a toxin, which triggers a pro-carcinogenic effect, to induce colon tumorigenesis [40]. In addition, *Proteobacteria*, as pro-inflammatory intestinal microbes, can multiply in the gut in response to an imbalanced microbial composition and are associated with disease occurrence and development [41]. During this experiment, astaxanthin intervention was found to significantly reverse the ethanol-induced increases in *Bacteroidetes* and *Proteobacteria*, restoring their proportions to the levels of the ND group. This indicates that the protective effect of astaxanthin is likely associated with its anti-inflammatory activity.

*Cyanobacteria* was completely suppressed in mice after treatment with ethanol as compared to the ethanol-free treatment. Although the abundance of *Cyanobacteria* is extremely low in the microbiota, we focused on this phylum because *Cyanobacteria* is completely inhibited in the gut when the diet contains ethanol, either with or without astaxanthin. This phenomenon indicated that astaxanthin did not affect the action of alcohol.

Accumulating reports have focused on the beneficial effects of *Akkermansia* on host metabolism. *Akkermansia* is a dominant genus in *Verrucomicrobia* and can degrade intestinal mucin [42], increasing mucus thickness and enhancing gut barrier function, which correlates inversely with the incidence of inflammation [43] and metabolic syndrome [18]. Lack of *Akkermansia* has been determined to be an early marker of alcohol-induced gut dysbiosis [37]. Moreover, ethanol exposure reduces the abundance of *Akkermansia* in both mice and humans, and the status of AFLD can be improved by oral supplementation of the genus, directly demonstrating the protective effect of this bacterium in AFLD [44]. As a probiotic, the abundance of *Akkermansia* can be affected by the administration of specific dietary components [45]. In the current research, the abundance of *Akkermansia* in ethanol-exposed AFLD mice dramatically reduced, and astaxanthin intervention obviously recovered its abundance, to even higher than that of the ND group. Our data suggest that mice with AFLD might benefit from astaxanthin supplementation increasing *Akkermansia*. Studies have proved that *Akkermansia* can play a protective role in ethanol-induced liver injury as a result of its function of improving the gut barrier [46], and the present research requires the further investigation of astaxanthin barrier function in the future.

The different bacterial species in the same genus may reflect different responses by the same treatment. Thus, it is indispensable to identify changes in the microbiota at the species level. In the present study, among the 13 OTUs altered by ethanol and reversed by astaxanthin intervention, the proportions of *Akkermansia muciniphila* (OTU36578) were significantly decreased in the Et group, while they were enriched in the EtAST and ND groups. *Akkermansia muciniphila* is a species of the *Akkermansia* genus, and extensive research has shown that it can improve the status of obesity, diabetes, and inflammation [47]. On the other hand, the species from *Butyricimonas* (OTU 61092, OTU22852, and OTU43831), S24-7 (OTU52622, OTU52789, OTU67103, OTU4618, and OTU11187), *Oscillospira* (OTU9917), *Clostridiales* (OTU60609 and OTU71112), and *Bilophila* (OTU2261) were significantly increased by ethanol diet and markedly reversed by astaxanthin supplementation. It has been reported that the abundance of *Oscillospira* and *Clostridales* increased in inflammatory response and is associated with the barrier injury of intestinal mucosa [48,49]. Stanislawski et al. demonstrated that *Bilophila* was positively correlated with the fat fraction of the liver [50]. Our research found that astaxanthin treatment significantly decreased the levels of ALT, AST, TG, LDL, and the index of LW/BW, which were increased by ethanol diet. Among the 13 OTUs altered by ethanol and reversed by astaxanthin supplementation, Spearman’s correlation analysis suggested that *Akkermansia muciniphila*, enriched in the EtAST group, was negatively associated with the AFLD phenotype. On the other hand, the 12 enriched OTUs in the Et group were positively associated with the AFLD phenotype. These results indicate that astaxanthin relieves the AFLD phenotype through its ability to enrich the bacteria responsible for intestinal integrity and anti-inflammation.

The typical characteristic of AFLD is excessive hepatic lipid accumulation. Therefore, lipid metabolic regulation plays an important role in the pathogenesis of metabolic disorders. In this study, the predicted metabolic function of the gut microbiota showed that the lipid metabolism pathway was increased after astaxanthin treatment, while further research proved that *Akkermansia* acts as a probiotic to promote lipid metabolism and avoid lipid excessive accumulation [51]. The results indicate that the inhibition by astaxanthin of excessive lipid accumulation in the liver may be associated with gut bacteria that promote lipid metabolism. In addition, astaxanthin increased the xenobiotic biodegradation and metabolism pathways. Xenobiotics are a kind of foreign chemical in living systems, which, after entering an organism, may induce adverse or even very serious consequences [52]. The liver is the major organ of metabolism, and astaxanthin supplementation can increase the abundance of bacteria that promote hepatic xenobiotic biodegradation and metabolism, resulting in detoxification and liver injury protection. However, the current findings need to be verified in larger samples.

Interestingly, astaxanthin was more effective in ameliorating alcohol-induced liver injury rather than that related to HFD. There is evidence that astaxanthin can protect against liver damage conditions like NAFLD and AFLD through multiple mechanisms, including antioxidant and anti-inflammatory effects [10,22,53]. In this research, mice in the Con group fed a high-fat diet did not show obvious inflammation, as measured by the liver index, histopathology, and liver gene expression. However, ethanol interference induced a marked inflammatory response which promoted astaxanthin to exert its anti-inflammatory effects to protect the liver from injury. This can also be explained from another aspect; specifically, astaxanthin will not disturb the metabolic balance under non-inflammatory conditions [54], which is consistent with the result that most responses in the AST group were similar to those in the Con group in our research. Future research should determine the content of astaxanthin in the mouse (liver and serum) and its feces.

## 5. Conclusions

Our research explored the protective effects of astaxanthin on AFLD injury mice and its regulation of gut microbiota. Astaxanthin has the ability to reverse the ethanol-induced liver weight ratio increase, hepatic inflammation, and lipid dysmetabolism. Furthermore, the overall structure and composition of the gut microbiota altered by ethanol feeding can be balanced by astaxanthin, particularly recovering the beneficial bacterium *Akkermansia*. Our results indicate that *Akkermansia* may be a potential target for the astaxanthin-related alleviation of AFLD, which provides further evidence for its molecular mechanism, and suggests that it could be used for the treatment of bacterial disorders induced by AFLD.

## Supplementary Materials

The following are available online at http://www.mdpi.com/2072-6643/10/9/1298/s1, Table S1: Compositions of the diets (DOC); Table S2: Primer sequences used for q-PCR reactions (DOC); Table S3: The OTUs obtained from high-throughput 16S rRNA gene sequencing (XLS); Figure S1: Rationality of sequencing data was evaluated by rarefaction and rank abundance curve (DOC 359); Figure S2: Cluster analysis using the UPGMA method based on unweighted Unifrac and weighted Unifrac analysis (DOC); Figure S3: The overall classification levels based on GraphlAn and LEfSe analyses were used to visualize the taxonomy compositions and bacterial abundance; Figure S4: Heatmap of the abundance of bacteria at the genus level at different groups (DOC); Figure S5: Astaxanthin protect ethanol-induced *Akkermansia muciniphila* depletion (DOC); Figure S6: Predicted functions for the altered metagenome of gut microbiota (PDF).

## Abbreviations

AST	astaxanthin
AFLD	alcoholic fatty liver disease
IL-1α	interleukin-1 alpha
MIP-2	macrophage inflammatory protein 2
ND	normal diet
Et	ethanol
ALT	alanine aminotransferase
TG	triacylglycerol
LDL	low-density lipoprotein
LW	liver weight
BW	body weight
Con	control
PCoA	principle coordinate analysis
OTUs	operational taxonomic units
KEGG	Kyoto Encyclopedia of Genes and Genomes
