# Supplementary material for: Astaxanthin Prevents Alcoholic Fatty Liver Disease by Modulating Mouse Gut Microbiota
Source: Nutrients. 2018 Sep 13;10(9):1298. doi: 10.3390/nu10091298 (PMC6164583; doi:10.3390/nu10091298)
Supplement: Supplementary file 1 [file nutrients-10-01298-s001.zip › Supplements/Table S1.docx]

Table S1. Compositions of the diets.

|  | ND | Con | AST | Et | EtAST |
| --- | --- | --- | --- | --- | --- |
|  | *wt %* | | | | |
| Feed | standard diet | high-fat liquid diets | | | |
| Astaxanthin [mg/kg bw] | - | - | 50.0 | - | 50.0 |
| Ethanol | - | - | - | 5.0 | 5.0 |
|  | *KJ %* | | | | |
| Fat | 12.3 | 35.0 | 35.0 | 35.0 | 35.0 |
| Protein | 24.4 | 18.0 | 18.0 | 18.0 | 18.0 |
| Carbohydrate | 63.3 | 47.0 | 47.0 | 11.0 | 11.0 |
| Ethanol | - | - | - | 36.0 | 36.0 |
| Energy content [KJ g^-1^] | 13.8 | 18.5 | 18.5 | 18.5 | 18.5 |

Diets were supplied by TROPHIC Animal Feed High-Tech Co., Ltd. Abbreviations: ND, normal diet group; Con, high-fat diet group; AST, astaxanthin supplied group; Et, 5 % ethanol supplied group; EtAST, 5 % ethanol plus astaxanthin supplied group.
