# Supplementary material for: Astaxanthin Prevents Alcoholic Fatty Liver Disease by Modulating Mouse Gut Microbiota
Source: Nutrients. 2018 Sep 13;10(9):1298. doi: 10.3390/nu10091298 (PMC6164583; doi:10.3390/nu10091298)
Supplement: Supplementary file 1 [file nutrients-10-01298-s001.zip › Supplements/Table S2.docx]

**Table S3. Primers sequences used in qRT-PCR analysis.**

| \| Gene  name \| \| --- \| | Forward primer (5’–3’ ) | Reverse primer (5’–3’ ) | Accession No. |
| --- | --- | --- | --- | --- |
| MIP-2 | GAAGTCATAGCCACTCTCAAGG | CTTCCGTTGAGGGACAGC | NM_009140 |
| IL-1α | TGCAGTCCATAACCCATGATC | ACAAACTTCTGCCTGACGAG | NM_010554 |
