# Supplementary figures and images for: Astaxanthin Prevents Alcoholic Fatty Liver Disease by Modulating Mouse Gut Microbiota
Source: Nutrients. 2018 Sep 13;10(9):1298. doi: 10.3390/nu10091298 (PMC6164583; doi:10.3390/nu10091298)

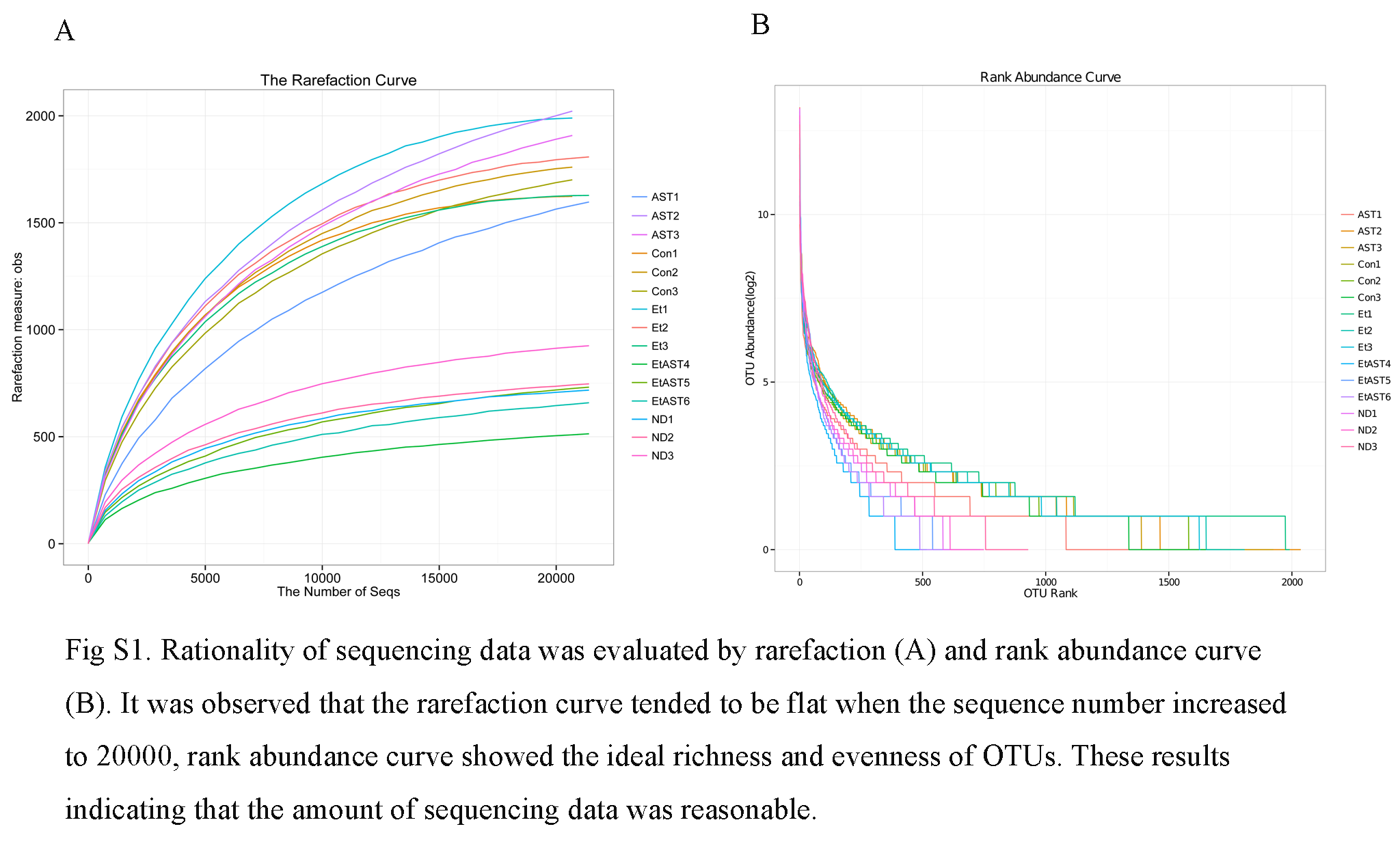

Supplement: Supplementary file 1 [file nutrients-10-01298-s001.zip › Supplements/fig S1.tif]

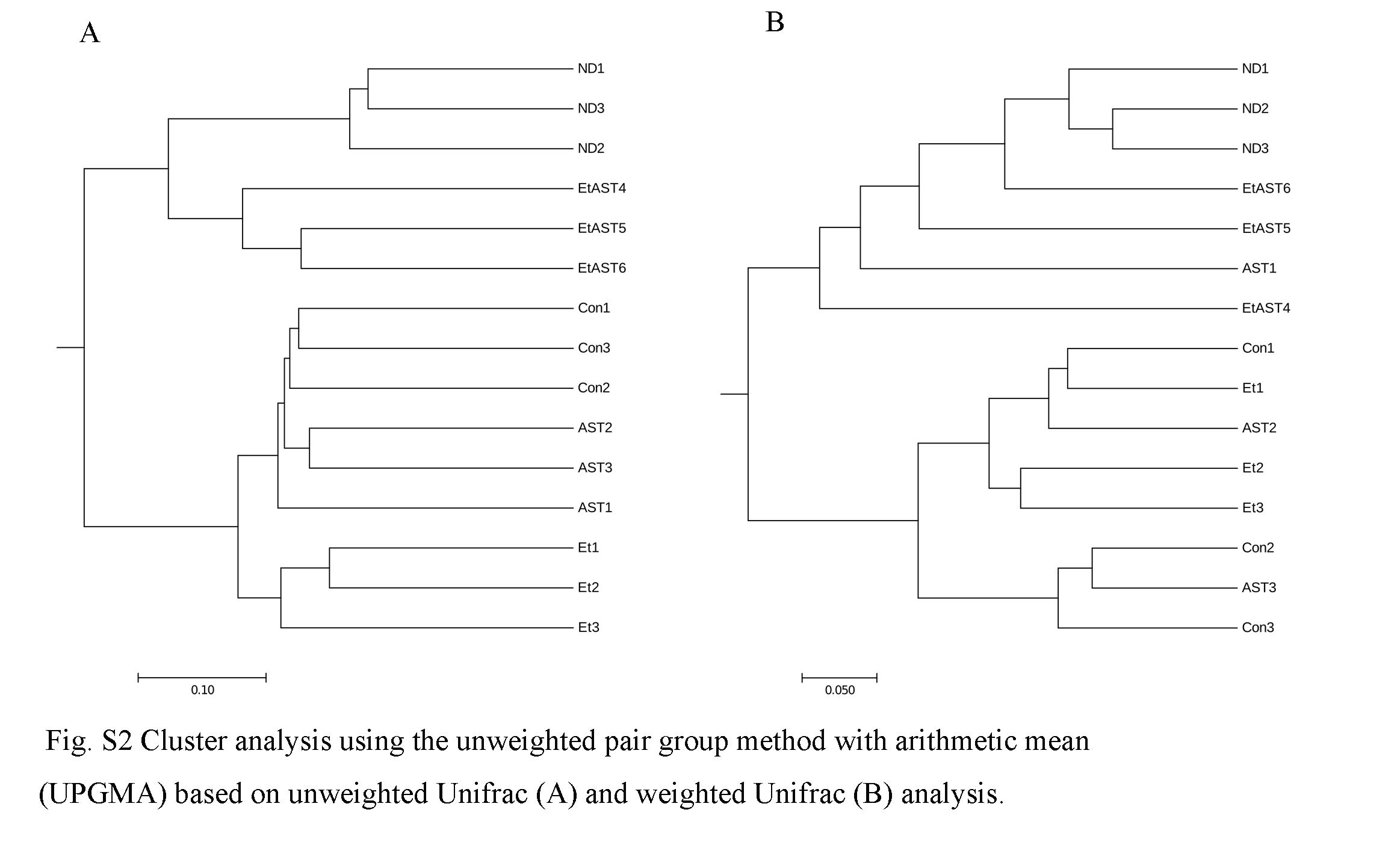

Supplement: Supplementary file 1 [file nutrients-10-01298-s001.zip › Supplements/Fig S2.tif]

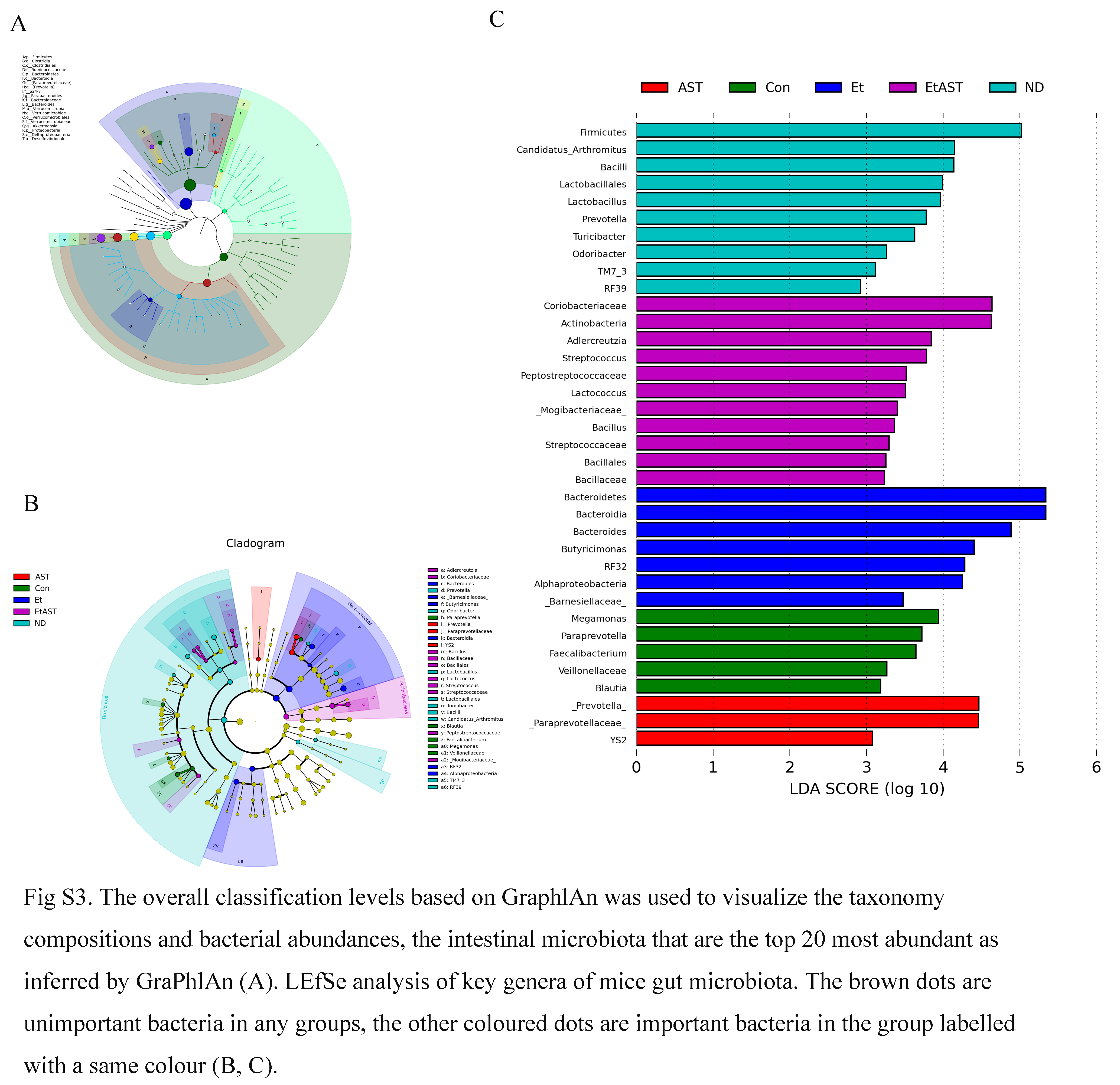

Supplement: Supplementary file 1 [file nutrients-10-01298-s001.zip › Supplements/Fig S3.tif]

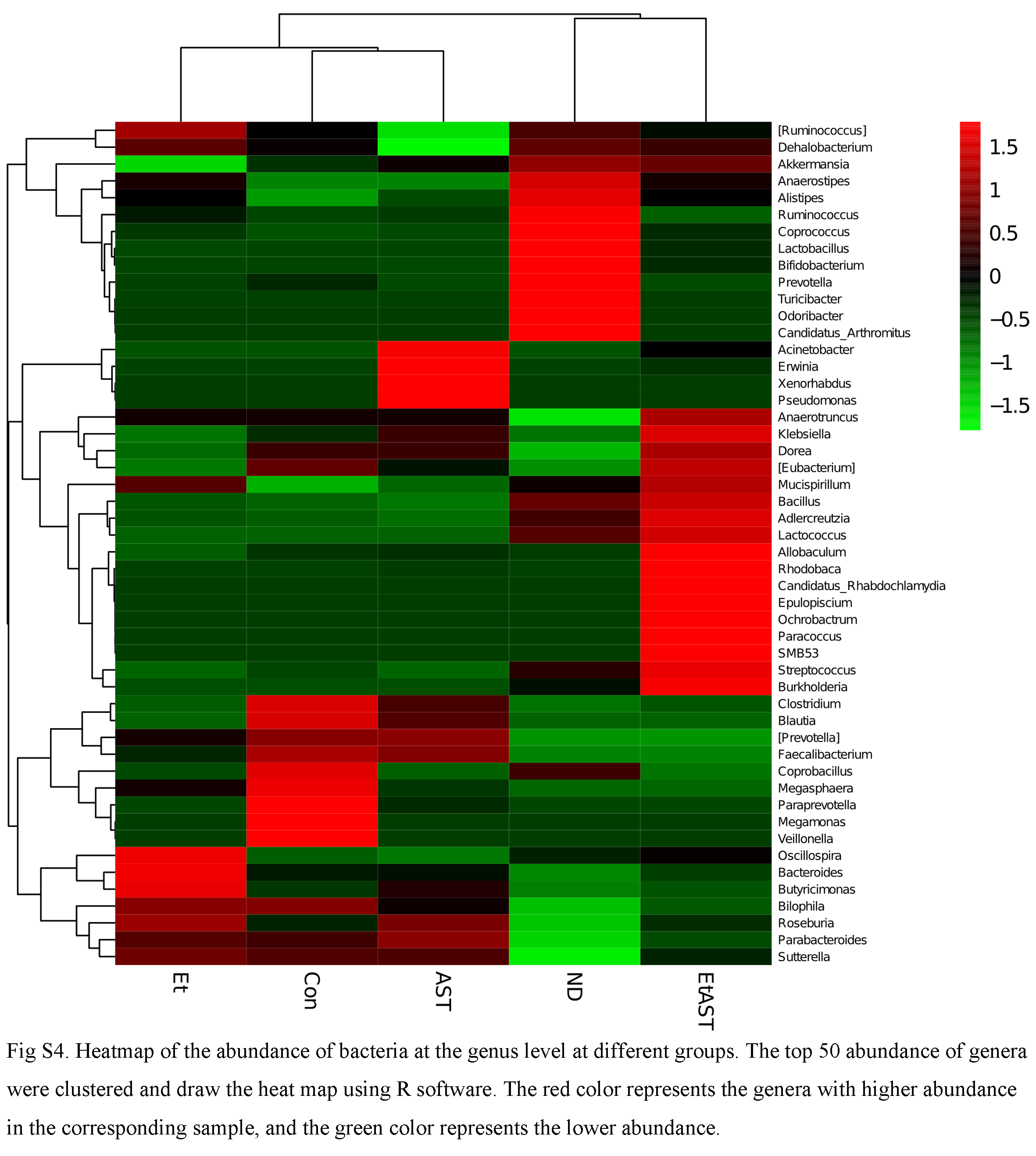

Supplement: Supplementary file 1 [file nutrients-10-01298-s001.zip › Supplements/Fig S4.tif]

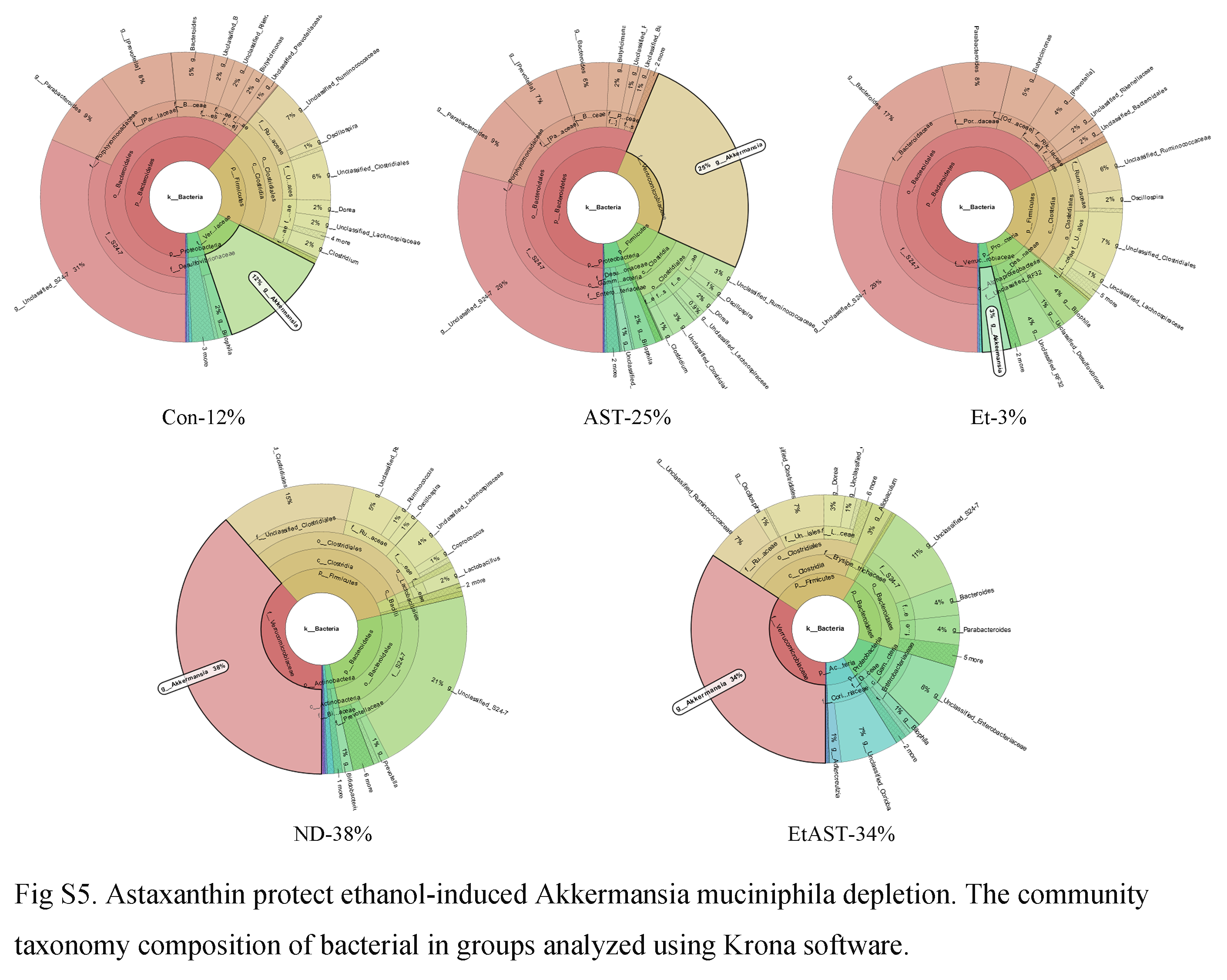

Supplement: Supplementary file 1 [file nutrients-10-01298-s001.zip › Supplements/Fig S5.tif]

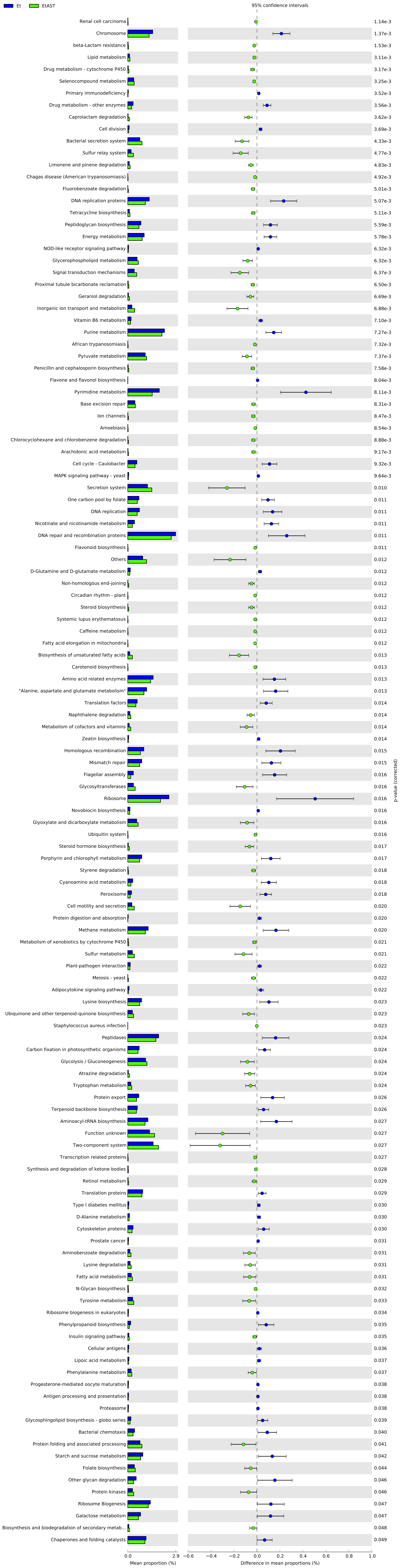

Supplement: Supplementary file 1 [file nutrients-10-01298-s001.zip › Supplements/Fig S6.pdf]
